# Accuracy of implants placed with CAD-CAM surgical templates using metal or peek sleeve: an *in vitro* analysis

**DOI:** 10.1590/0103-644020256696

**Published:** 2026-06-26

**Authors:** Silvio Mecca, Mateus Alves Mecca, Christian Rado Jarry, Marcelo Henrique Napimoga

**Affiliations:** 1 Implantology Department, Faculdade São Leopoldo Mandic, Instituto São Leopoldo Mandic, Campinas, Brazil; 2 Dental School, University of São Paulo, São Paulo, Brazil; 3 Laboratory of Neuroimmune Interface of Pain Research, Faculdade São Leopoldo Mandic, Instituto São Leopoldo Mandic, Campinas, Brazil

**Keywords:** Sleeves, guided implant surgery, accuracy

## Abstract

The sleeve material in printed surgical guides directly influences the accuracy of guided implant surgery, yet its impact on the 3D position of implants remains unclear. This study evaluated the effect of metal and PEEK (Polyether ether ketone) sleeves on implant accuracy compared to planned virtual positioning. Using CT scans and virtual planning software (coDiagnostiX), a STRAUMANN® RC BLT 4.1 x 10 mm implant was planned in the element 36 region of a partially edentulous patient (Ethical approval #5.879.848). Twenty surgical guides of hemi-arch were printed (10 with metal sleeves, 10 with PEEK sleeves) and tested using a master model. Implants were inserted through static guides, and the positional deviations were analyzed by comparing planned versus inserted implants using angular, coronal, and apical metrics. Statistical analysis showed significantly lower angular deviation in the PEEK sleeve group (57.2% lower than metal sleeves, p < 0.001), indicating greater precision. However, coronal deviation was significantly higher in the PEEK group (66.3% higher than metal sleeves, p = 0.006). No significant differences were found in apical deviation (p = 0.186). These findings suggest that the choice of sleeve material significantly impacts guided surgery accuracy, with PEEK sleeves offering superior angular precision but higher coronal deviation compared to metal sleeves.

## Resumo

O material das anilhas em guias cirúrgicos impressos influencia diretamente a precisão da cirurgia guiada por implantes, embora seu impacto na posição tridimensional (3D) dos implantes ainda não esteja totalmente claro. Este estudo avaliou o efeito das anilhas metálicas e de PEEK (Poliéter-éter-cetona) na precisão da instalação dos implantes em comparação com o planejamento virtual. Utilizando tomografias computadorizadas e software de planejamento virtual (coDiagnostiX), foi planejado um implante STRAUMANN® RC BLT 4.1 x 10 mm na região do elemento 36 de um paciente parcialmente edêntulo (Aprovação ética nº 5.879.848). Vinte guias cirúrgicos de hemi-arco foram impressos (10 com anilhas metálicas e 10 com anilhas de PEEK) e testados em um modelo mestre. Os implantes foram inseridos por meio de guias estáticos, e as variações posicionais foram analisadas comparando os implantes planejados com os inseridos, utilizando métricas angulares, coronais e apicais. A análise estatística mostrou uma diferença angular significativamente menor no grupo com anilhas de PEEK (57,2% menor do que no grupo com buchas metálicas, p < 0,001), indicando maior precisão. No entanto, a variação coronal foi significativamente maior no grupo com PEEK (66,3% maior do que no grupo com anilhas metálicas, p = 0,006). Não foram encontradas diferenças significativas na variação apical (p = 0,186). Esses achados sugerem que a escolha do material da anilhas impacta significativamente a precisão da cirurgia guiada, sendo que as anilhas de PEEK oferecem maior precisão angular, mas apresentam maior desvio coronal em comparação com as metálicas.

**Figure:**
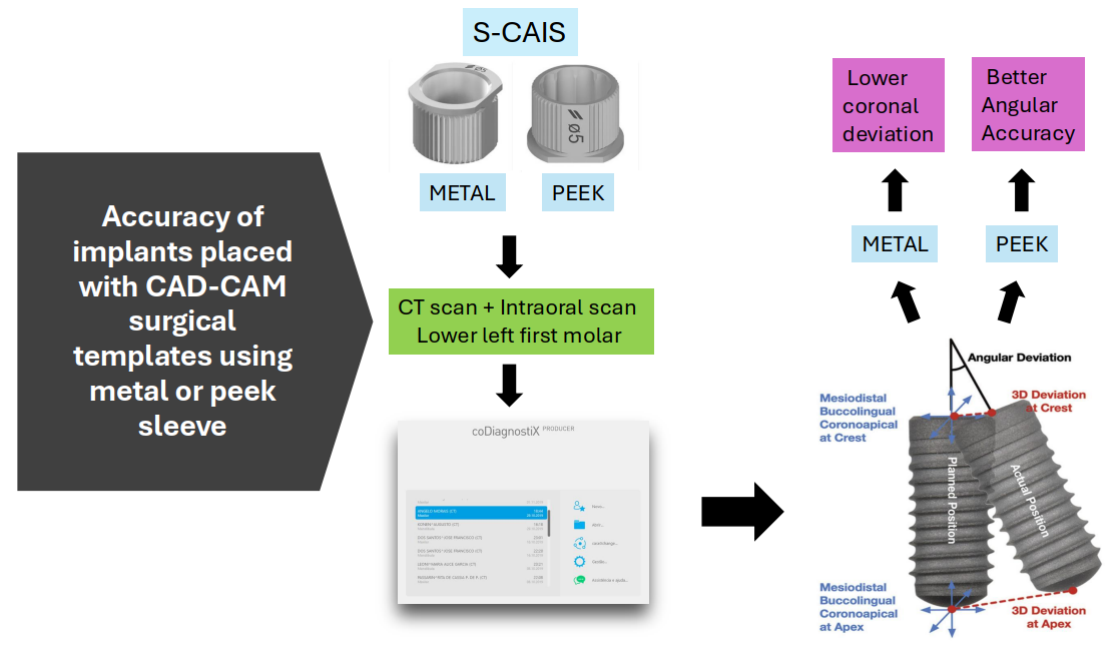

## Introduction

Currently marketed implants have high survival rates and clinical success ^1^ ^,^ ^2^ ^,^ ^3^. For this reason, the primary focus of research has shifted from osseointegration to the correct and optimal positioning of the implant. The correct 3D position of the implant is a prerequisite for an excellent aesthetic result, while poor positioning increases the risk of complications, with estimates suggesting that nearly half of peri-implantitis cases are due to this reason ^4^ ^,^ ^5^.

Surgical guides reduce the uncertainties inherent in freehand or conventional surgery ^6^. Guided surgery systems are classified as dynamic or static. Dynamic guides offer real-time visualization during surgery but are less accurate than the static system, in addition to having higher financial costs ^7^ ^,^ ^8^. Static guides offer better accuracy in most clinical situations. They can be printed or milled, supported on bone, mucosa, or teeth, and are obtained through the acquisition of a CT scan and a scan of oral structures, which are then overlaid and planned using CAD software for virtual surgery ^9^ ^,^ ^10^ ^,^ ^11^ ^,^ ^12^ ^,^ ^13^.

In addition to optimizing the three-dimensional position of implants, static guides also offer other advantages, such as reducing surgical damage, improving postoperative recovery, reducing patient discomfort, aiding in immediate function, causing less trauma, and assisting in bone grafts and surgeries in atrophic ridges ^13^.

However, several factors can directly influence the accuracy of surgical guides, which may be present either individually or in combination, such as errors in image acquisition or processing, the fit accuracy of the surgical guide, types of 3D printers or resins, milling, variation in sleeve height, type of sleeve, and movement of the guide during the surgical process ^14^.

It is common in the literature to find studies comparing the accuracy of printed versus milled guides or comparing the various materials used for these guides. In printed static surgical guide systems, there is a sequence of events for their fabrication, and we can consider a high potential for what we call isolated or combined mechanical errors, ranging from the printing of the guide, installation of sleeves, and the final finishing of these parts. The assembly of the guide is exclusively manual and can interfere with the accuracy of the final planned implant position ^15^.

Sleeves are devices that are manually fitted into the hole of the printed guide. They are responsible for fitting the handles that guide and stabilize a sequence of drills for bone perforation during surgery and for final installation through a specific driver. The most significant clinical errors that can occur during surgery include movement of the guide as a whole, gaps between the handle and sleeve, gaps between the drill and handle, and gaps between the implant driver and sleeve.

An important aspect to be studied is the movement of the guide during surgery, especially during the use of handles, which are fitted into the sleeve to reduce the diameter of the drills and are held by the operator during drilling. Sleeves can be made of metal or PEEK. When the operator uses the PEEK sleeve system, the internal design of this device is different from the metal one, whose function is to provide a locking of the handle on the sleeve, eliminating the need for the operator to hold the handle during surgery, which can avoid possible unwanted movements. For this reason, the PEEK sleeve is classified as "Self Locking” ^16^.

Most sleeves currently used are made of metal, which is why it has been proposed to research different materials to try to reduce errors and misfits of the guided surgery kit instruments in these devices. Although some studies in the literature address the use of surgical guides, there are few studies comparing different types of sleeves. Therefore, it is important to study this topic and identify whether there will be an influence on the accuracy of static surgical guides. Therefore, the objective of this laboratory study is to evaluate the effect of using two types of sleeves (metal and PEEK) in static surgical guides, and whether there will be any interference in the accuracy of the installed implants compared to the planned virtual positioning. The null hypothesis is that different sleeves do not interfere with the accuracy of installed implants when compared with the planned virtual positioning.

## Material and methods

**Figure 1:**
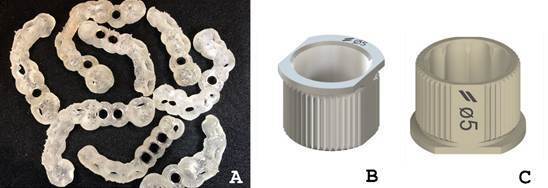
(A) Printed surgical guides; (B) Metal sleeve; (C) PEEK sleeve (Adapted from Straumann).

This is an in vitro study, reported in accordance with the recommendations of the Revised Standards for Quality Improvement Reporting Excellence (SQUIRE 2.0) tool, from the EQUATOR network. A CT scan and an intraoral scan of a partially edentulous patient (missing the lower left first molar) were used (Ethical approval #5.879.848). The DICOM and STL files were imported into a virtual planning software (coDiagnostiX 9.12; Dental Wings), and a Straumann RC Bone Level Tapered implant (Straumann) was digitally inserted into the mandibular prosthetic space (element 36). The surgical guide was designed and exported in STL format. Twenty ^20^ surgical guides of hemi-arch tooth-supported were printed, with 0.15-mm offset and 3-mm thickness (Cosmos SG, Yller- Neodent®, Brazil) (Figure 1A) using a DLP 3D printer (Flashforge Hunter) and divided into two groups: 10 with metal sleeves (Figure 1B) and 10 with PEEK sleeves (Figure 1C) (Straumann). The sample size (n = 10) of each group was based on a previous study ^14^.

**Figure 2:**
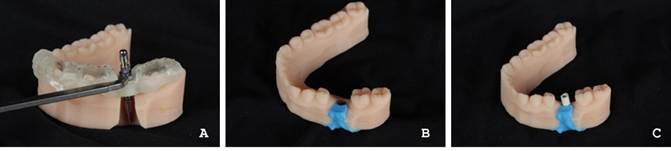
(A): Implant positioned; (B): Addition of silicone-based impression material; (C): Scanbody positioned.

The patient’s intraoral scan was printed in high-precision resin for dental models (Cosmos Dental Model DLP, Yller-Neodent®, Brazil), and a cut was made in the region of element 36 corresponding to the implant body to allow the surgical guide to fit and the passive installation of the implant through the different sleeves. To eliminate the variable “surgical act” or drilling process by the operator on the model, the implant was fixed using light addition silicone (VPS Impression Light, Yller- Neodent®, Brazil). No adjustments were necessary to fit the surgical templates (Figure 2A- C).

**Figure 3:**
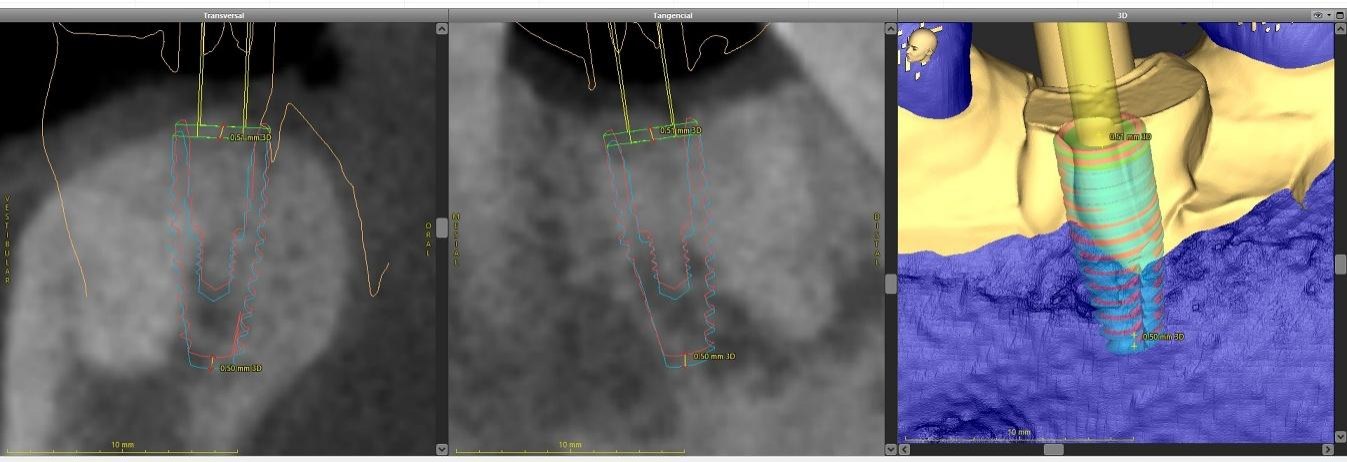
Final implant position (blue) overlaid with virtually planned position (red). A: Cross-sectional view; (B) Tangential view; (C) 3D view.

A Straumann Scanbody was inserted using light finger pressure over each implant, and the model was scanned using an intraoral scanner (Primescan, Sirona Dentsply) (Figure 2C). The generated STL file was imported into the coDiagnostiX 9.12 software and registered with the existing virtual planning, using the function “Verification Tool” (Figure 3), allowing the comparison between the planned implant position and the actual implant inserted into the master model, performed automatically by the software. The deviations studied were angular, apical (3D apex offset), and coronal (3D entry point offset).

### Statistical analysis

The data were checked for normal distribution and homoscedasticity, identifying that the values for angular and apical deviation had non-normal distribution and heteroscedasticity, respectively. Thus, comparisons between the groups with surgical guides using metal sleeves and PEEK sleeves were made using Student's t-test for independent samples or Mann-Whitney tests. Statistical calculations were performed using SPSS 23 (SPSS Inc.), with a significance level set at 5%.

## Results

**Figure 4:**
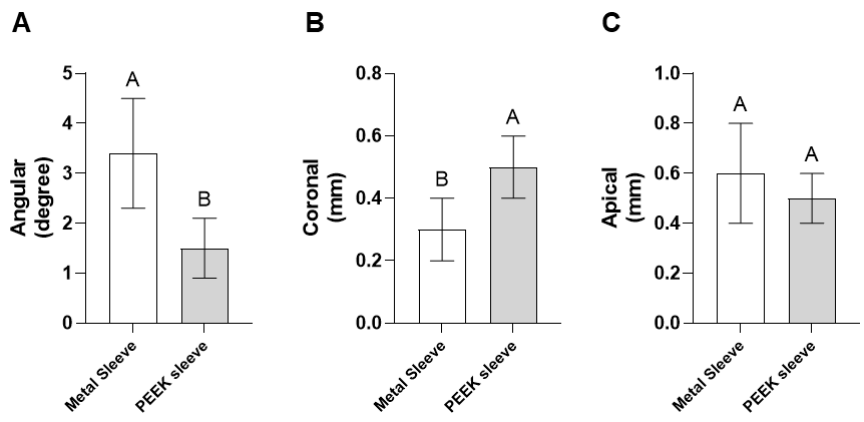
Implants installed with surgical guides planned and printed by the CAD-CAM system using metal or PEEK sleeves. Data present mean (± standard deviation) of Angular Deviation (A), Coronal deviation (B), and Apical deviation (C). Different uppercase letters above the columns indicate a statistically significant difference between groups.

The angular deviation of implants installed using surgical guides planned and printed through the CAD-CAM system was significantly lower in the group with PEEK sleeves (p <.001), being, on average, 57.2% lower than that observed in the group with metal sleeves (Figure 4A). On the other hand, it was found that implants installed using guides with PEEK sleeves had a significantly greater coronal deviation compared to the group with metal sleeves (p=.006) (Figure 4B). The coronal deviation (3D entry poin affset) was, on average, 66.3% higher in the PEEK sleeve group. As for apical deviation (3D apex offset), there was no significant difference (p 0.186) in the measured values between the groups whose implants were installed using guides with metal and PEEK sleeves (Figure 4C). All values are also detailed as demonstrated in Table 1.

**Table 1:** Means (standard deviations), medians, and minimum and maximum values of angular, coronal, and apical deviations of implants placed using a surgical guide planned and printed with the CAD-CAM system, with metallic and PEEK sleeves.

Deviation	Guide with Sleeve	
	Metallic (T-Sleeve)	PEEK (Self-Locking)
Angular (degrees)		
*Mean (standard deviation)*	3,4 ^B^ (1,1)	1,5 ^A^ (0,6)
*Median*	3,4	1,5
*Minimum / Maximum values*	2,2; 4,7	0,6; 2,4
Coronal (mm)		
*Mean (standad deviation)*	0,3 ^A^ (0,1)	0,5 ^B^ (0,1)
*Median*	0,3	0,5
*Minimum / maximum values*	0,1; 0,5	0,3; 0,7
Apical (mm)		
*Mean (standard deviation)*	0,6 ^A^ (0,2)	0,5 ^A^ (0,1)
*Median*	0,7	0,5
*Minimum / Maximum values*	0,3; 0,9	0,4; 0,7

## Discussion

Surgical guides, given the variables required for their production, must demonstrate precise in loco fit, and another critical factor is the precision of the sleeve fit within the guide, which will transfer all the planning information to the correct 3D positioning of the implant. This study found significant differences between the two types of sleeves used, which impacted the outcome regarding angular deviation. Therefore, the null hypothesis was not accepted.

The type of sleeve used should be considered when executing cases, and whether the implant system offers such an option. Particularly in borderline cases, PEEK sleeves may be considered the first choice. Surgical guides have various factors that can influence their accuracy, from the type of printer used to other production methods like milling. Additionally, the post-printing processing and ensuring that the hole for sleeve installation is adequate are crucial so that the sleeve is installed passively and in a way that respects the printed slot design, ensuring that the sleeve is neither too far forward nor too far back in its positioning ^14^.

In this study, the use of PEEK sleeves showed better results when comparing the planned and executed implant positions, varying from 0.6º to 2.4º, with a mean of 1.5º (±0.6) in angular deviation, which critically influences the 3D positioning of the implant, the biomechanics of the installed element, periodontal health, minimizing peri-implantitis, and, consequently, its longevity ^5^ ^,^ ^17^. On the other hand, PEEK sleeves showed a significantly greater coronal or entry deviation than metal sleeves, with a mean of 0.5 mm (±0.1). These deviations demonstrate the difficulty of the passive fitting and settling of the PEEK sleeve in the surgical guide by the operator during the final preparation and adjustment of the surgical guide. However, this value is clinically acceptable, as it falls within the average error of the guided system, with 1.2 mm for coronal deviation as presented by the third EAO conference ^18^. These deviations may be related to the intrinsic mechanical behavior of PEEK, a semi-crystalline thermoplastic with lower rigidity compared to metal sleeves, which can slightly deform under pressure during surgical guide positioning. Moreover, these deviations demonstrate the difficulty of achieving an entirely passive fit and proper seating of the PEEK sleeve into the printed guide during the final preparation and adjustment by the operator.

Together, these factors may explain the higher coronal deviation observed for PEEK sleeves, despite their favorable angular precision. There were no significant statistical differences in apical deviation, with values ranging from 0.3 mm to 0.7 mm. These results for the final implant position fall within the proposed safety zone of 2 mm ^19^.

Coronal and apical deviations are related to the three-dimensional positioning of the implant's entry and apex, respectively. The greater coronal deviation observed with PEEK sleeves may be attributed to the difficulty in achieving proper seating of these sleeves in the surgical guide, which can lead to variation in the entry point, particularly in the vertical direction. On the other hand, the greater apical deviation associated with metal sleeves may be explained by a more pronounced angular deviation, resulting in a larger three-dimensional discrepancy at the implant apex. The deviations can be related to several factors, such as guide printing, guide fit, the internal spacing of the sleeve, the spacing and geometry of the guide hole for receiving the sleeve, drill length, and the acquisition of digital files ^17^ ^,^ ^19^.

Digitally guided implant surgery protocols show significantly higher levels of accuracy compared to conventional techniques. The use of this technology enhances the precision of implant placement in the correct 3D position. Most studies agree that the entirely digitally guided protocol is the gold standard in clinical practice ^6^ ^,^ ^20^. The angular deviations in computer-guided surgeries are not influenced by the surgical experience of the dental professionals ^21^. Regarding the behavior of patients undergoing computer-guided surgery, levels of anxiety and postoperative pain showed no statistical differences compared to the conventional process, reflecting the normal healing process of intraoral wounds ^22^.

This study was conducted to eliminate the "surgical drilling" variable by creating a cutout in the master model for passive implant installation. However, this influenced the trajectory of the bed that should have been prepared. The authors adopted this method due to the limitation of reproducing the conditions of actual bone density and the manipulation of the contra-angle for bone drilling; therefore, it may not reflect the actual clinical scenario ^23^. The accuracy results for PEEK sleeves were superior to those of metal sleeves; however, more controlled clinical trials are needed to clarify the influence of different materials used in the production of these devices.

Based on the results of this in vitro study, we concluded that the planned and final position of the implant guided through the static surgical guide was influenced by the sleeve material type, and PEEK sleeves demonstrated greater accuracy with significantly lower angular deviation than the metal sleeve group.

## Data Availability

The research data are available upon request

## References

[1] De Angelis F, Papi P, Mencio F, Rosella D, Di Carlo S, Pompa G (2017). Implant survival and success rates in patients with risk factors: Results from a long-term retrospective study with a 10 to 18 years follow-up. Eur Rev Med Pharmacol Sci.

[2] Derks J, Håkansson J, Wennström JL, Tomasi C, Larsson M, Berglundh T (2015). Effectiveness of implant therapy analyzed in a Swedish population: Early and late implant loss. J Dent Res.

[3] Van Velzen FJ, Ofec R, Schulten EA, Ten Bruggenkate CM (2015). 10-year survival rate and the incidence of peri-implant disease of 374 titanium dental implants with a SLA surface: A prospective cohort study in 177 fully and partially edentulous patients. Clin Oral Implants Res.

[4] Tahmaseb A, Wismeijer D, Coucke W, Derksen W (2014). Computer technology applications in surgical implant dentistry: A systematic review. Int J Oral Maxillofac Implants.

[5] Canullo L, Tallarico M, Radovanovic S, Delibasic B, Covani U, Rakic M (2016). Distinguishing predictive profiles for patient-based risk assessment and diagnostics of plaque-induced, surgically and prosthetically triggered peri-implantitis. Clin Oral Implants Res.

[6] Varga E, Antal M, Major L, Kiscsatári R, Braunitzer G, Piffkó J (2020). Guidance means accuracy: A randomized clinical trial on freehand versus guided dental implantation. Clin Oral Implants Res.

[7] Jung RE, Schneider D, Ganeles J, Wismeijer D, Zwahlen M, Hämmerle CH (2009). Computer technology applications in surgical implant dentistry: A systematic review. Int J Oral Maxillofac Implants.

[8] Younes F, Cosyn J, De Bruyckere T, Cleymaet R, Bouckaert E, Eghbali A (2018). A randomized controlled study on the accuracy of freehanded, pilot-drill guided and fully guided implant surgery in partially edentulous patients. J Clin Periodontol.

[9] Behneke A, Burwinkel M, Behneke N (2012). Factors influencing transfer accuracy of cone beam CT-derived template-based implant placement. Clin Oral Implants Res.

[10] Ersoy AE, Turkyilmaz I, Ozan O, McGlumphy EA (2008). Reliability of implant placement with stereolithographic surgical guides generated from computed tomography: Clinical data from 94 implants. J Periodontol.

[11] Nickenig HJ, Wichmann M, Hamel J, Schlegel KA, Eitner S (2010). Evaluation of the difference in accuracy between implant placement by virtual planning data and surgical guide templates versus the conventional freehand method: A combined in vivo-in vitro technique using cone-beam CT (Part II). J Craniomaxillofac Surg.

[12] Ozan O, Orhan K, Turkyilmaz I (2011). Correlation between bone density and angular deviation of implants placed using CT-generated surgical guides. J Craniofac Surg.

[13] Hämmerle CH, Stone P, Jung RE, Kapos T, Brodala N (2009). Consensus statements and recommended clinical procedures regarding computer-assisted implant dentistry. Int J Oral Maxillofac Implants.

[14] Herschdorfer L, Negreiros WM, Gallucci GO, Hamilton A (2021). Comparison of the accuracy of implants placed with CAD-CAM surgical templates manufactured with various 3D printers: An in vitro study. J Prosthet Dent.

[15] Chen L, Lin WS, Polido WD, Eckert GJ, Morton D (2019). Accuracy, reproducibility, and dimensional stability of additively manufactured surgical templates. J Prosthet Dent.

[16] Ozan O, Şeker E, Çakmak G, Guo X, Yilmaz B (2022). Effect of guide sleeve material, region, diameter, and number of times drills were used on the material loss from sleeves and drills used for surgical guides: An in vitro study. J Prosthet Dent.

[17] Tahmaseb A, Wu V, Wismeijer D, Coucke W, Evans C (2018). The accuracy of static computer-aided implant surgery: A systematic review and meta-analysis. Clin Oral Implants Res.

[18] Sicilia A, Botticelli D (2012). Working Group 3. Computer-guided implant therapy and soft- and hard-tissue aspects: The Third EAO Consensus Conference 2012. Clin Oral Implants Res.

[19] Wismeijer D, Joda T, Flügge T, Fokas G, Tahmaseb A, Bechelli D (2018). ITI Consensus Report: Digital technologies. Clin Oral Implants Res.

[20] Khaohoen A, Powcharoen W, Sornsuwan T, Chaijareenont P, Rungsiyakull C, Rungsiyakull P (2024). Accuracy of implant placement with computer-aided static, dynamic, and robot-assisted surgery: A systematic review and meta-analysis of clinical trials. BMC Oral Health.

[21] De Almeida JC, Soares MQS, Mamani MP, Franco A, Junqueira JLC (2024). Influence of surgeon experience on implant placement in guided surgeries: A systematic review and meta-analysis of randomized clinical trials. J Prosthet Dent.

[22] Kunavisarut C, Santivitoonvong A, Chaikantha S, Pornprasertsuk-Damrongsri S, Joda T (2022). Patient-reported outcome measures comparing static computer-aided implant surgery and conventional implant surgery for single-tooth replacement: A randomized controlled trial. Clin Oral Implants Res.

[23] Sun Y, Ding Q, Tang L, Zhang L, Sun Y, Xie Q (2019). Accuracy of a chairside fused deposition modeling 3D-printed single-tooth surgical template for implant placement: An in vitro comparison with a light cured template. J Craniomaxillofac Surg.

